# Facts Worth Remembering

**Published:** 1880-01

**Authors:** 


### FACTS WORTH REMEMBERING.

Sudden Deaths do not come from heart disease, one case in twenty, but from congestion of the lungs or brain, or from apoplexy. More die from congestion of the lungs than of the brain, and more of congestion of the brain than from apoplexy.

Sudden death from heart disease is usually caused by rupture of some large artery near the heart ; from congestion of the lungs, by instantly stopping the breath ; from congestion of the brain, by causing pressure on the brain, which paralyzes and instantly destroys life ; from apoplexy, by hemorrhage in the brain.

Heart disease most frequently results from neglected or improperly-treated rheumatism. It more often follows mild rheumatism than the severe kind, because severe rheumatism receives prompt treatment, while the mild form is often neglected and left to work its way to the heart.

Persons who suppose themselves suffering from heart disease, because they have pain in the region of the heart, or palpitation, seldom have any disease of that organ. In nine cases out of ten, they are sufferers from dyspepsia—nothing more. Congestion of the lungs is most frequently caused by a sudden change from the heat of an ill-ventilated room or railroad car, or street car, to the cold air outside, without being protected by sufficient clothing ; hence, many persons thus seized drop dead in the streets.

Congestion of the brain most frequently results from trouble and anxiety of mind, producing sleeplessness, followed by engorgement of the small blood vessels of the brain, sudden loss of vital power, and almost instant death. Apoplexy may be an inherited disease, or it may be induced by too free living, or its opposite, too great abstemiousness. Paralysis may affect only a small portion of the body, from a finger or toe to an entire limb, or it may disable half the body, or the whole body, when death soon follows. When half the body is affected by paralysis, we may be certain that the seat of the disease is in the opposite side of the brain, because nerve fibres cross. Partial paralysis is often temporary when caused by the rupture of a small blood vessel, if the clot is got rid of by absorption or otherwise.

Although this is a disease that all classes of people are liable to, its most destructive work is done among the depraved and dissipated. There is no doubt that the habitual use of tobacco is one of the most prominent causes of paralysis and other nerve diseases.

